# Examination of 189 *Campylobacter* Species Isolates from the Global Enteric Multicenter Study

**DOI:** 10.1128/MRA.00646-20

**Published:** 2020-07-23

**Authors:** Riley G. Risteen, Tracy H. Hazen, Jane M. Michalski, Frédéric Poly, Sharon M. Tennant, David A. Rasko

**Affiliations:** a Institute for Genome Sciences, University of Maryland School of Medicine, Baltimore, Maryland, USA; b Department of Microbiology and Immunology, University of Maryland School of Medicine, Baltimore, Maryland, USA; c Enteric Diseases Department, Naval Medical Research Center, Silver Spring, Maryland, USA; d Center for Vaccine Development and Global Health, University of Maryland School of Medicine, Baltimore, Maryland, USA; Georgia Institute of Technology

## Abstract

We have examined the draft genomes of 189 *Campylobacter* species isolates from the Global Enteric Multicenter Study, in which *Campylobacter* species were identified as significant pathogens.

## ANNOUNCEMENT

Diarrheal disease is one of the most significant causes of morbidity and death in lower- and middle-income countries (1, 2). The Global Enteric Multicenter Study (GEMS), a prospective case-control study unprecedented in scale, was undertaken to examine the causes of acute moderate-to-severe diarrhea in children under the age of 5 years at seven sites in Africa and Asia (3, 4). The *Campylobacter* species isolates were cultured as described previously (5). Briefly, *Campylobacter* species were identified by plating diluted stool onto *Campylobacter* blood agar plates overnight at 37°C. Single colonies were examined, and those that were catalase and oxidase positive were examined microscopically for small Gram-negative rods that were slightly curved or S-shaped. A total of 189 *Campylobacter* species isolates were examined by whole-genome sequencing (Table 1). In this submission, we have sequenced 35 isolates of Campylobacter coli, 3 isolates of Campylobacter lari, and 151 isolates of Campylobacter jejuni. These isolates represent a selection of isolates from both cases and controls from each of the seven sites of the GEMS.

**TABLE 1:** Genome statistics

Sample	Species	Country of origin	No. of reads	No. of bases sequenced	Genome coverage (×)	No. of contigs	Size (bp)	GC content (%)	*N*_50_ (bp)	Whole-genome sequence accession no.	SRA accession no.
100179	*Campylobacter jejuni*	Gambia	6,203,710	936,760,210	540.5	38	1,733,171	30.47	286,964	JAAJZR000000000	SRR11268331
100854	*Campylobacter jejuni*	Gambia	8,906,498	1,344,881,198	789.3	42	1,703,935	30.55	176,129	JAAJZQ000000000	SRR11268330
102163	*Campylobacter jejuni*	Gambia	15,812,146	2,387,634,046	1,436.9	41	1,661,608	30.8	157,957	JAAJZP000000000	SRR11268329
102389	*Campylobacter jejuni*	Gambia	7,434,154	1,122,557,254	642.5	46	1,747,259	30.47	219,778	JAAJZO000000000	SRR11268327
102405	*Campylobacter jejuni*	Gambia	4,066,296	614,010,696	362.8	37	1,692,235	30.28	118,378	JAAJZN000000000	SRR11268326
102406	*Campylobacter jejuni*	Gambia	4,778,384	721,535,984	423.4	41	1,704,044	30.34	154,532	JAAJZM000000000	SRR11268325
102470	*Campylobacter jejuni*	Gambia	7,069,274	1,067,460,374	662.3	35	1,611,762	30.68	181,371	JAAJZL000000000	SRR11268324
102495	*Campylobacter jejuni*	Gambia	3,337,740	503,998,740	297.1	39	1,696,560	30.27	117,403	JAAJZK000000000	SRR11268323
102500	*Campylobacter jejuni*	Gambia	6,173,594	932,212,694	581.6	23	1,602,957	30.48	298,201	JAAJZJ000000000	SRR11268322
102624	*Campylobacter jejuni*	Gambia	5,794,162	874,918,462	468.3	57	1,868,440	31.53	154,560	JAAJZI000000000	SRR11268321
102796	*Campylobacter jejuni*	Gambia	4,272,412	645,134,212	374.1	31	1,724,717	30.29	181,874	JAAJZH000000000	SRR11268320
102808	*Campylobacter jejuni*	Gambia	6,356,930	959,896,430	558.2	24	1,719,584	30.29	286,960	JAAJZG000000000	SRR11268319
102878	*Campylobacter jejuni*	Gambia	5,740,342	866,791,642	498.0	36	1,740,422	30.26	154,555	JAAJZF000000000	SRR11268318
102879	*Campylobacter jejuni*	Gambia	5,483,538	828,014,238	447.6	144	1,849,863	30.21	37,456	JAAJZE000000000	SRR11268316
102895	*Campylobacter jejuni*	Gambia	5,547,986	837,745,886	474.9	47	1,763,939	30.13	175,306	JAAJZD000000000	SRR11268315
102920	*Campylobacter jejuni*	Gambia	4,479,356	676,382,756	404.8	33	1,670,818	30.27	179,731	JAAJZC000000000	SRR11268314
102928	*Campylobacter jejuni*	Gambia	6,007,354	907,110,454	523.7	49	1,732,203	30.29	126,828	JAAJZB000000000	SRR11268313
102930	*Campylobacter jejuni*	Gambia	5,696,706	860,202,606	499.9	34	1,720,905	30.29	181,871	JAAJZA000000000	SRR11268312
102954	*Campylobacter jejuni*	Gambia	5,149,276	777,540,676	448.0	47	1,735,550	30.29	119,613	JAAJYZ000000000	SRR11268311
102958	*Campylobacter jejuni*	Gambia	4,729,804	714,200,404	413.5	21	1,727,062	30.27	286,964	JAAJYY000000000	SRR11268310
103022	*Campylobacter jejuni*	Gambia	6,206,532	937,186,332	561.7	37	1,668,488	30.28	179,745	JAAJYX000000000	SRR11268309
103067	*Campylobacter jejuni*	Gambia	4,389,078	662,750,778	376.0	43	1,762,822	30.14	260,015	JAAJYW000000000	SRR11268308
103092	*Campylobacter jejuni*	Gambia	4,759,248	718,646,448	419.3	32	1,713,942	30.2	179,602	JAAJYV000000000	SRR11268307
103169	*Campylobacter jejuni*	Gambia	4,669,524	705,098,124	409.6	27	1,721,614	30.29	290,964	JAAJYU000000000	SRR11268305
103223	*Campylobacter jejuni*	Gambia	5,748,660	868,047,660	492.4	45	1,763,047	30.14	183,776	JAAJYT000000000	SRR11268304
103290	*Campylobacter jejuni*	Gambia	4,386,094	662,300,194	385.9	24	1,716,368	30.26	154,555	JAAJYS000000000	SRR11268303
103392	*Campylobacter jejuni*	Gambia	3,276,204	494,706,804	293.6	26	1,685,246	30.27	178,894	JAAJYR000000000	SRR11268302
120722	*Campylobacter jejuni*	Gambia	6,315,044	953,571,644	590.9	25	1,613,725	30.52	151,343	JAAJYQ000000000	SRR11268301
201108	*Campylobacter coli*	Mali	7,545,542	1,139,376,842	667.0	37	1,708,305	31.53	220,782	JAAKBA000000000	SRR11268291
202143	*Campylobacter jejuni*	Mali	7,256,954	1,095,800,054	625.5	34	1,751,776	30.19	179,603	JAAJYP000000000	SRR11268300
202370	*Campylobacter jejuni*	Mali	7,754,042	1,170,860,342	652.1	38	1,795,460	30.42	227,186	JAAJYO000000000	SRR11268299
202376	*Campylobacter jejuni*	Mali	7,148,482	1,079,420,782	670.1	14	1,610,873	30.51	289,842	JAAJYN000000000	SRR11268298
202388	*Campylobacter jejuni*	Mali	7,688,784	1,161,006,384	676.1	43	1,717,214	30.52	188,083	JAAJYM000000000	SRR11268297
202648	*Campylobacter jejuni*	Mali	7,446,846	1,124,473,746	644.3	28	1,745,343	30.2	221,009	JAAJYL000000000	SRR11268296
202813	*Campylobacter jejuni*	Mali	7,559,292	1,141,453,092	642.9	43	1,775,560	30.41	179,602	JAAJYK000000000	SRR11268294
202823	*Campylobacter coli*	Mali	7,093,986	1,071,191,886	621.3	85	1,724,140	31.5	38,132	JAAKAZ000000000	SRR11268290
202982	*Campylobacter coli*	Mali	7,647,166	1,154,722,066	636.7	181	1,813,673	31.43	24,698	JAAKAY000000000	SRR11268270
203005	*Campylobacter coli*	Mali	7,203,060	1,087,662,060	630.5	29	1,725,153	31.56	228,869	JAAKAX000000000	SRR11268259
203008	*Campylobacter coli*	Mali	6,361,554	960,594,654	550.2	36	1,745,999	31.46	135,201	JAAKAW000000000	SRR11268328
203010	*Campylobacter coli*	Mali	6,071,568	916,806,768	513.0	139	1,787,086	31.96	33,582	JAAKAV000000000	SRR11268317
203047	*Campylobacter jejuni*	Mali	8,280,092	1,250,293,892	720.9	87	1,734,318	30.69	66,924	JAAJYJ000000000	SRR11268293
203061	*Campylobacter jejuni*	Mali	7,907,742	1,194,069,042	717.5	67	1,664,323	30.69	74,708	JAAJYI000000000	SRR11268292
203065	*Campylobacter jejuni*	Mali	6,275,160	947,549,160	539.9	51	1,755,162	30.43	179,754	JAAJYH000000000	SRR11268209
203086	*Campylobacter jejuni*	Mali	7,593,766	1,146,658,666	690.7	31	1,660,160	30.62	167,636	JAAJYG000000000	SRR11268208
203109	*Campylobacter coli*	Mali	5,126,262	774,065,562	448.7	37	1,724,961	31.52	217,565	JAAKAU000000000	SRR11268306
203122	*Campylobacter jejuni*	Mali	5,595,104	844,860,704	494.7	19	1,707,883	30.36	154,544	JAAJYF000000000	SRR11268207
203138	*Campylobacter coli*	Mali	6,718,906	1,014,554,806	594.1	20	1,707,836	31.31	218,038	JAAKAT000000000	SRR11268295
203174	*Campylobacter jejuni*	Mali	6,560,894	990,694,994	596.4	24	1,661,016	30.65	288,875	JAAJYE000000000	SRR11268206
203196	*Campylobacter jejuni*	Mali	4,312,172	651,137,972	377.3	45	1,725,848	30.18	116,451	JAAJYD000000000	SRR11268205
203266	*Campylobacter jejuni*	Mali	9,282,290	1,401,625,790	776.6	62	1,804,710	30.93	148,393	JAAJYC000000000	SRR11268204
203429	*Campylobacter jejuni*	Mali	7,183,880	1,084,765,880	624.7	107	1,736,430	30.93	40,389	JAAJYB000000000	SRR11268203
203531	*Campylobacter jejuni*	Mali	6,471,590	977,210,090	590.4	26	1,655,127	30.42	151,233	JAAJYA000000000	SRR11268201
203542	*Campylobacter jejuni*	Mali	6,579,938	993,570,638	584.7	42	1,699,349	30.53	140,624	JAAJXZ000000000	SRR11268200
203645	*Campylobacter coli*	Mali	8,250,328	1,245,799,528	737.2	51	1,689,940	31.62	111,902	JAAKAS000000000	SRR11268202
203720	*Campylobacter jejuni*	Mali	6,627,054	1,000,685,154	588.4	19	1,700,696	30.26	188,108	JAAJXY000000000	SRR11268199
203813	*Campylobacter jejuni*	Mali	4,344,186	655,972,086	365.0	47	1,797,234	30.13	188,749	JAAJXX000000000	SRR11268198
203873	*Campylobacter coli*	Mali	3,742,778	565,159,478	335.0	21	1,686,902	31.31	163,658	JAAKAR000000000	SRR11268175
203875	*Campylobacter jejuni*	Mali	4,140,734	625,250,834	354.8	48	1,762,279	30.15	154,426	JAAJXW000000000	SRR11268197
203886	*Campylobacter jejuni*	Mali	7,068,262	1,067,307,562	623.2	26	1,712,631	30.65	329,559	JAAJXV000000000	SRR11268196
203886_2	*Campylobacter jejuni*	Mali	5,717,342	863,318,642	509.0	14	1,696,040	30.43	327,588	JAAJTZ000000000	SRR11268275
203890	*Campylobacter jejuni*	Mali	3,193,368	482,198,568	266.9	38	1,806,461	30.13	160,806	JAAJXU000000000	SRR11268195
203927	*Campylobacter jejuni*	Mali	5,618,672	848,419,472	471.9	218	1,797,735	30.18	20,329	JAAJXT000000000	SRR11268194
203928	*Campylobacter coli*	Mali	4,261,520	643,489,520	375.5	44	1,713,910	30.2	109,394	JAAJXS000000000	SRR11268177
203953	*Campylobacter coli*	Mali	7,149,212	1,079,531,012	572.7	78	1,884,894	31.18	131,021	JAAKAQ000000000	SRR11268193
204026	*Campylobacter coli*	Mali	6,894,468	1,041,064,668	607.4	41	1,714,090	31.69	171,977	JAAKAP000000000	SRR11268182
204028	*Campylobacter coli*	Mali	7,559,840	1,141,535,840	657.7	135	1,735,644	31.5	32,193	JAAKAO000000000	SRR11268158
204061	*Campylobacter jejuni*	Mali	5,335,324	805,633,924	460.2	31	1,750,476	30.23	157,758	JAAJXR000000000	SRR11268176
204097	*Campylobacter coli*	Mali	10,420,228	1,573,454,428	887.7	85	1,772,455	31.21	183,172	JAAJXQ000000000	SRR11268174
204115	*Campylobacter jejuni*	Mali	5,970,620	901,563,620	511.9	156	1,761,268	31.28	26,471	JAAKAN000000000	SRR11268256
204137	*Campylobacter jejuni*	Mali	3,680,086	555,692,986	303.8	44	1,828,955	30.15	125,137	JAAJXP000000000	SRR11268173
204171	*Campylobacter jejuni*	Mali	4,842,366	731,197,266	430.8	13	1,697,378	30.46	220,957	JAAJXO000000000	SRR11268172
204177	*Campylobacter jejuni*	Mali	4,815,226	727,099,126	428.2	19	1,697,996	30.46	171,489	JAAJXN000000000	SRR11268171
204211	*Campylobacter jejuni*	Mali	3,415,020	515,668,020	305.0	35	1,690,989	30.44	199,496	JAAJXM000000000	SRR11268170
204252	*Campylobacter coli*	Mali	4,367,558	659,501,258	398.9	18	1,653,505	30.65	199,497	JAAJXL000000000	SRR11268169
204269	*Campylobacter jejuni*	Mali	3,676,862	555,206,162	326.0	30	1,703,046	31.34	133,203	JAAKAM000000000	SRR11268245
204304	*Campylobacter jejuni*	Mali	9,000,690	1,359,104,190	819.1	35	1,659,322	30.92	321,859	JAAJXK000000000	SRR11268168
204332	*Campylobacter jejuni*	Kenya	4,031,530	608,761,030	359.4	30	1,693,869	30.4	197,610	JAAJXJ000000000	SRR11268167
400636	*Campylobacter jejuni*	Kenya	7,710,952	1,164,353,752	649.8	46	1,791,802	30.33	230,736	JAAJXI000000000	SRR11268166
400738	*Campylobacter jejuni*	Kenya	5,121,988	773,420,188	445.3	85	1,736,727	30.23	83,710	JAAJXH000000000	SRR11268165
400745	*Campylobacter jejuni*	Kenya	4,946,518	746,924,218	432.0	42	1,728,967	30.21	148,462	JAAJXG000000000	SRR11268192
400787	*Campylobacter jejuni*	Kenya	7,438,370	1,123,193,870	645.7	119	1,739,435	30.46	29,731	JAAJXF000000000	SRR11268191
400797	*Campylobacter jejuni*	Kenya	6,124,418	924,787,118	544.7	40	1,697,839	30.49	139,022	JAAJXE000000000	SRR11268190
401051	*Campylobacter jejuni*	Kenya	3,314,158	500,437,858	293.5	36	1,705,014	30.23	179,526	JAAJXD000000000	SRR11268189
401125	*Campylobacter jejuni*	Kenya	4,314,926	651,553,826	386.9	32	1,684,238	30.22	293,998	JAAJXC000000000	SRR11268188
401207	*Campylobacter jejuni*	Kenya	7,113,334	1,074,113,434	634.6	34	1,692,512	30.29	140,280	JAAJXB000000000	SRR11268187
401272	*Campylobacter jejuni*	Kenya	4,766,168	719,691,368	425.0	30	1,693,542	30.31	170,153	JAAJXA000000000	SRR11268186
401419	*Campylobacter jejuni*	Kenya	9,693,016	1,463,645,416	840.1	63	1,742,226	30.75	153,642	JAAJWZ000000000	SRR11268185
401554	*Campylobacter jejuni*	Kenya	4,126,962	623,171,262	369.6	27	1,685,911	30.28	148,420	JAAJWY000000000	SRR11268184
402330	*Campylobacter jejuni*	Kenya	7,350,650	1,109,948,150	657.6	28	1,687,804	30.54	291,465	JAAJWX000000000	SRR11268183
402665	*Campylobacter jejuni*	Kenya	3,394,254	512,532,354	302.0	27	1,696,910	30.2	224,886	JAAJWW000000000	SRR11268181
402692	*Campylobacter jejuni*	Kenya	4,168,684	629,471,284	357.8	46	1,759,362	30.15	119,983	JAAJWV000000000	SRR11268180
402718	*Campylobacter jejuni*	Kenya	7,688,018	1,160,890,718	675.0	84	1,719,910	30.5	66,365	JAAJWU000000000	SRR11268179
402747	*Campylobacter jejuni*	Kenya	4,463,534	673,993,634	392.8	25	1,715,706	30.25	154,554	JAAJWT000000000	SRR11268178
402930	*Campylobacter jejuni*	Kenya	4,735,142	715,006,442	433.3	11	1,650,263	30.5	181,931	JAAJWS000000000	SRR11268164
403073	*Campylobacter coli*	Kenya	3,330,874	502,961,974	313.6	25	1,603,818	30.55	189,338	JAAKBC000000000	SRR11268234
403107	*Campylobacter jejuni*	Kenya	1,668,904	252,004,504	152.1	26	1,657,349	31.36	141,482	JAAKAL000000000	SRR11268223
403132	*Campylobacter jejuni*	Kenya	4,606,780	695,623,780	411.1	52	1,692,195	30.27	110,293	JAAJWR000000000	SRR11268163
403138	*Campylobacter jejuni*	Kenya	4,837,712	730,494,512	436.3	28	1,674,409	30.34	291,943	JAAJWQ000000000	SRR11268162
403205	*Campylobacter jejuni*	Kenya	4,879,426	736,793,326	457.2	20	1,611,600	30.43	180,161	JAAJWP000000000	SRR11268161
403259	*Campylobacter jejuni*	Kenya	5,342,188	806,670,388	502.1	42	1,606,710	30.47	141,357	JAAJWO000000000	SRR11268160
403459	*Campylobacter jejuni*	Kenya	5,669,500	856,094,500	499.7	161	1,713,238	30.29	30,234	JAAJWN000000000	SRR11268159
403772	*Campylobacter jejuni*	Kenya	5,181,170	782,356,670	424.8	78	1,841,742	31.9	109,555	JAAJWM000000000	SRR11268157
403834	*Campylobacter jejuni*	India	6,305,490	952,128,990	549.9	26	1,731,379	30.22	189,394	JAAJWL000000000	SRR11268156
500153	*Campylobacter jejuni*	India	6,803,370	1,027,308,870	570.7	44	1,800,234	30.42	179,937	JAAJWK000000000	SRR11268155
500213	*Campylobacter jejuni*	India	5,345,888	807,229,088	494.3	19	1,633,002	30.63	296,986	JAAJWJ000000000	SRR11268154
500215	*Campylobacter jejuni*	India	6,673,956	1,007,767,356	603.0	40	1,671,227	30.69	184,448	JAAJWI000000000	SRR11268153
500340	*Campylobacter jejuni*	India	6,408,974	967,755,074	533.7	71	1,813,151	30.7	129,061	JAAJWH000000000	SRR11268152
500380	*Campylobacter jejuni*	India	6,333,678	956,385,378	548.6	36	1,743,323	30.36	157,821	JAAJWG000000000	SRR11268340
500400	*Campylobacter jejuni*	India	7,287,656	1,100,436,056	609.8	65	1,804,682	30.86	183,643	JAAJWF000000000	SRR11268339
500435	*Campylobacter jejuni*	India	5,905,760	891,769,760	487.3	33	1,830,172	30.13	155,314	JAAJWE000000000	SRR11268338
500438	*Campylobacter jejuni*	India	5,402,998	815,852,698	496.1	26	1,644,604	30.52	195,816	JAAJWD000000000	SRR11268257
500461	*Campylobacter jejuni*	India	9,498,782	1,434,316,082	816.2	47	1,757,345	30.67	169,666	JAAKBB000000000	SRR11268212
500480	*Campylobacter jejuni*	India	5,512,630	832,407,130	485.0	44	1,716,146	30.33	183,127	JAAJWC000000000	SRR11268255
500481	*Campylobacter jejuni*	India	4,762,896	719,197,296	422.4	24	1,702,587	30.26	179,580	JAAJWB000000000	SRR11268254
500496	*Campylobacter jejuni*	India	4,474,008	675,575,208	420.6	18	1,606,157	30.46	185,577	JAAJWA000000000	SRR11268253
500499	*Campylobacter jejuni*	India	4,306,534	650,286,634	405.1	22	1,605,363	30.46	289,666	JAAJVZ000000000	SRR11268252
500563	*Campylobacter jejuni*	India	3,799,978	573,796,678	326.7	26	1,756,195	30.33	183,645	JAAJVY000000000	SRR11268251
500570	*Campylobacter jejuni*	India	3,790,534	572,370,634	306.9	52	1,865,128	29.94	135,995	JAAJVX000000000	SRR11268250
500591	*Campylobacter jejuni*	India	3,856,054	582,264,154	340.1	50	1,712,101	30.21	106,391	JAAJVW000000000	SRR11268249
500592	*Campylobacter jejuni*	India	3,250,106	490,766,006	280.9	28	1,746,970	30.33	183,643	JAAJVV000000000	SRR11268248
500656	*Campylobacter jejuni*	India	4,044,558	610,728,258	361.0	21	1,691,876	30.33	197,545	JAAJVU000000000	SRR11268247
500704	*Campylobacter jejuni*	India	5,176,274	781,617,374	466.3	22	1,676,235	30.28	286,518	JAAJVT000000000	SRR11268246
500708	*Campylobacter jejuni*	India	4,080,866	616,210,766	348.8	25	1,766,601	30.23	185,888	JAAJVS000000000	SRR11268244
500710	*Campylobacter lari*	India	5,000,046	755,006,946	446.5	18	1,690,895	30.33	286,415	JAAJVR000000000	SRR11268243
503734	*Campylobacter jejuni*	India	6,220,854	939,348,954	638.7	37	1,470,770	29.98	127,023	JAAJTY000000000	SRR11268274
503824	*Campylobacter jejuni*	India	6,596,446	996,063,346	586.0	31	1,699,640	30.49	183,845	JAAJVQ000000000	SRR11268242
504218	*Campylobacter lari*	India	6,764,224	1,021,397,824	666.9	51	1,531,487	30.01	142,803	JAAJTX000000000	SRR11268273
504478	*Campylobacter lari*	India	6,995,424	1,056,309,024	707.3	37	1,493,515	29.9	143,751	JAAJTW000000000	SRR11268272
504557	*Campylobacter jejuni*	India	5,937,750	896,600,250	520.6	171	1,722,124	30.53	24,430	JAAJVP000000000	SRR11268241
504611	*Campylobacter jejuni*	India	4,722,690	713,126,190	420.1	38	1,697,648	30.22	180,012	JAAJVO000000000	SRR11268240
504764	*Campylobacter jejuni*	India	4,280,046	646,286,946	362.6	42	1,782,268	30.19	180,046	JAAJVN000000000	SRR11268239
510942	*Campylobacter jejuni*	India	5,613,382	847,620,682	487.6	33	1,738,188	30.33	148,210	JAAJVM000000000	SRR11268238
511171	*Campylobacter jejuni*	India	3,872,784	584,790,384	336.9	67	1,735,885	30.18	128,079	JAAJVL000000000	SRR11268237
521136	*Campylobacter jejuni*	India	5,597,404	845,208,004	525.7	20	1,607,829	30.47	289,690	JAAJVK000000000	SRR11268236
521162	*Campylobacter jejuni*	India	5,551,710	838,308,210	478.2	70	1,752,915	30.27	64,603	JAAJVJ000000000	SRR11268235
600001	*Campylobacter coli*	Bangladesh	6,716,828	1,014,241,028	558.0	72	1,817,597	31.57	163,114	JAAKAK000000000	SRR11268281
600030	*Campylobacter jejuni*	Bangladesh	6,103,334	921,603,434	520.9	111	1,769,377	31.57	32,279	JAAKAJ000000000	SRR11268271
600032	*Campylobacter jejuni*	Bangladesh	7,212,888	1,089,146,088	593.8	59	1,834,224	30.39	174,893	JAAJVI000000000	SRR11268233
600034	*Campylobacter jejuni*	Bangladesh	8,838,754	1,334,651,854	755.6	92	1,766,375	30.53	52,978	JAAJVH000000000	SRR11268232
600099	*Campylobacter jejuni*	Bangladesh	4,802,490	725,175,990	436.5	16	1,661,406	30.46	153,905	JAAJVG000000000	SRR11268231
600883	*Campylobacter jejuni*	Bangladesh	5,279,702	797,235,002	459.6	32	1,734,451	30.23	119,457	JAAJVF000000000	SRR11268230
600978	*Campylobacter jejuni*	Bangladesh	3,440,852	519,568,652	293.2	44	1,772,006	30.21	158,690	JAAJVE000000000	SRR11268229
601037	*Campylobacter jejuni*	Bangladesh	7,135,232	1,077,420,032	644.4	23	1,671,899	30.38	129,032	JAAJVD000000000	SRR11268228
602950	*Campylobacter jejuni*	Bangladesh	6,987,878	1,055,169,578	644.2	90	1,638,017	30.61	39,830	JAAJVC000000000	SRR11268227
603180	*Campylobacter jejuni*	Bangladesh	6,059,946	915,051,846	531.1	48	1,723,063	30.38	103,103	JAAJVB000000000	SRR11268226
603771	*Campylobacter jejuni*	Bangladesh	2,884,482	435,556,782	260.9	36	1,669,130	30.32	217,195	JAAJVA000000000	SRR11268225
604313	*Campylobacter coli*	Bangladesh	4,858,970	733,704,470	391.7	83	1,872,995	30.07	64,463	JAAJUZ000000000	SRR11268224
604335	*Campylobacter coli*	Bangladesh	5,029,314	759,426,414	437.5	85	1,735,638	31.25	44,965	JAAKAI000000000	SRR11268269
604349	*Campylobacter jejuni*	Bangladesh	5,115,372	772,421,172	437.8	52	1,764,238	30.15	120,039	JAAJUY000000000	SRR11268222
604421	*Campylobacter jejuni*	Bangladesh	4,313,588	651,351,788	403.9	20	1,612,509	30.46	183,854	JAAJUX000000000	SRR11268221
604447	*Campylobacter jejuni*	Bangladesh	3,836,990	579,385,490	361.0	22	1,605,028	30.47	166,133	JAAJUW000000000	SRR11268220
621131	*Campylobacter jejuni*	Pakistan	5,314,150	802,436,650	458.0	36	1,752,159	30.27	158,756	JAAJUV000000000	SRR11268219
700462	*Campylobacter jejuni*	Pakistan	7,264,686	1,096,967,586	621.6	41	1,764,719	30.52	195,884	JAAJUU000000000	SRR11268218
700490	*Campylobacter jejuni*	Pakistan	6,778,386	1,023,536,286	592.6	34	1,727,095	30.56	183,122	JAAJUT000000000	SRR11268217
700544	*Campylobacter coli*	Pakistan	6,983,836	1,054,559,236	594.4	35	1,774,282	30.36	183,059	JAAJUS000000000	SRR11268216
703113	*Campylobacter coli*	Pakistan	8,323,440	1,256,839,440	720.4	35	1,744,622	31.46	162,987	JAAKAH000000000	SRR11268268
703171	*Campylobacter coli*	Pakistan	5,581,320	842,779,320	471.9	35	1,786,025	31.31	170,264	JAAKAG000000000	SRR11268267
703275	*Campylobacter coli*	Pakistan	6,374,972	962,620,772	567.9	40	1,694,913	31.58	148,597	JAAKAF000000000	SRR11268266
703313	*Campylobacter coli*	Pakistan	7,593,126	1,146,562,026	637.2	27	1,799,272	31.38	187,295	JAAKAE000000000	SRR11268265
703452	*Campylobacter coli*	Pakistan	5,086,318	768,034,018	408.4	170	1,880,625	31.28	25,602	JAAKAD000000000	SRR11268264
703460	*Campylobacter jejuni*	Pakistan	5,219,202	788,099,502	426.1	55	1,849,671	31.29	78,608	JAAKAC000000000	SRR11268263
703549	*Campylobacter jejuni*	Pakistan	4,151,542	626,882,842	388.4	20	1,613,952	30.46	183,854	JAAJUR000000000	SRR11268215
703550	*Campylobacter jejuni*	Pakistan	3,597,594	543,236,694	283.6	46	1,915,743	30.06	141,849	JAAJUQ000000000	SRR11268214
703552	*Campylobacter jejuni*	Pakistan	5,493,168	829,468,368	429.9	52	1,929,628	30.25	159,911	JAAJUP000000000	SRR11268213
703556	*Campylobacter jejuni*	Pakistan	1,715,182	258,992,482	160.6	20	1,612,458	30.46	204,239	JAAJUO000000000	SRR11268211
703559	*Campylobacter jejuni*	Pakistan	4,210,794	635,829,894	375.0	90	1,695,440	30.46	77,683	JAAJUN000000000	SRR11268210
703614	*Campylobacter jejuni*	Pakistan	5,558,436	839,323,836	476.9	25	1,759,887	30.33	153,853	JAAJUM000000000	SRR11268289
703615	*Campylobacter coli*	Pakistan	7,414,056	1,119,522,456	631.2	37	1,773,729	30.59	106,560	JAAJUL000000000	SRR11268288
703632	*Campylobacter coli*	Pakistan	5,720,462	863,789,762	476.6	36	1,812,223	31.29	169,567	JAAKAB000000000	SRR11268262
703637	*Campylobacter jejuni*	Pakistan	4,708,756	711,022,156	440.7	18	1,613,479	30.46	204,373	JAAJUK000000000	SRR11268287
703638	*Campylobacter coli*	Pakistan	3,762,142	568,083,442	338.3	66	1,679,162	30.42	108,950	JAAJUJ000000000	SRR11268286
703644	*Campylobacter coli*	Pakistan	6,563,838	991,139,538	528.8	99	1,874,173	31.94	142,499	JAAKAA000000000	SRR11268261
703646	*Campylobacter coli*	Pakistan	6,769,006	1,022,119,906	578.1	32	1,768,138	31.43	151,722	JAAJZZ000000000	SRR11268260
703661	*Campylobacter jejuni*	Pakistan	6,706,838	1,012,732,538	581.2	32	1,742,438	31.44	264,498	JAAJZY000000000	SRR11268258
703664	*Campylobacter coli*	Pakistan	1,962,430	296,326,930	183.9	33	1,611,531	30.47	118,642	JAAJUI000000000	SRR11268285
703709	*Campylobacter coli*	Pakistan	6,706,300	1,012,651,300	596.0	28	1,699,150	31.47	220,108	JAAJZX000000000	SRR11268337
703726	*Campylobacter coli*	Pakistan	8,427,844	1,272,604,444	637.2	147	1,997,094	31.23	56,886	JAAJZW000000000	SRR11268336
703890	*Campylobacter jejuni*	Pakistan	7,028,076	1,061,239,476	583.0	64	1,820,341	31.43	94,220	JAAJZV000000000	SRR11268335
704017	*Campylobacter coli*	Pakistan	5,840,460	881,909,460	513.4	31	1,717,949	30.37	173,578	JAAJUH000000000	SRR11268284
704042	*Campylobacter jejuni*	Pakistan	6,729,102	1,016,094,402	596.2	36	1,704,314	31.52	149,934	JAAJZU000000000	SRR11268334
704082	*Campylobacter jejuni*	Pakistan	5,120,440	773,186,440	451.2	27	1,713,608	30.32	185,957	JAAJUG000000000	SRR11268283
704083	*Campylobacter coli*	Pakistan	4,658,208	703,389,408	407.4	34	1,726,402	30.29	179,602	JAAJUF000000000	SRR11268282
704192	*Campylobacter coli*	Pakistan	7,532,250	1,137,369,750	618.6	55	1,838,551	31.32	117,002	JAAJZT000000000	SRR11268333
704216	*Campylobacter jejuni*	Pakistan	6,730,994	1,016,380,094	595.3	169	1,707,348	31.68	22,048	JAAJZS000000000	SRR11268332
704227	*Campylobacter jejuni*	Pakistan	5,271,176	795,947,576	449.1	39	1,772,240	30.33	165,219	JAAJUE000000000	SRR11268280
704230	*Campylobacter jejuni*	Pakistan	5,598,968	845,444,168	474.7	43	1,781,173	30.13	180,021	JAAJUD000000000	SRR11268279
704264	*Campylobacter jejuni*	Pakistan	5,665,686	855,518,586	488.8	124	1,750,245	30.19	31,635	JAAJUC000000000	SRR11268278
710603	*Campylobacter jejuni*	Pakistan	5,812,220	877,645,220	502.1	35	1,747,934	30.18	333,527	JAAJUB000000000	SRR11268277
721225	*Campylobacter jejuni*	Mali	2,932,018	442,734,718	254.4	30	1,740,017	30.19	155,321	JAAJUA000000000	SRR11268276

Genomic DNA was isolated from cultures grown overnight in lysogeny broth. DNA was extracted in a 96-well format from 100 μl of sample using the MagAttract PowerMicrobiome DNA/RNA kit (Qiagen, Hilden, Germany) automated on a Hamilton Microlab STAR robotic platform. Bead disruption was conducted with a TissueLyser II instrument (20 Hz for 20 min) in a 96-deep-well plate in the presence of 200 μl phenol-chloroform. Genomic DNA was eluted in 90 μl water after magnetic bead cleanup. The resulting genomic DNA was quantified using PicoGreen. The sequencing libraries were generated with the KAPA HyperPrep kit (catalog number KK8504) and sequenced on the Illumina NovaSeq platform using a 150-bp paired-end kit.

The total number of reads generated for each isolate is listed in Table 1; values averaged 1,725,208 bp per genome. All software was used with default values. Raw sequencing reads were filtered to remove contaminating PhiX reads using BBDuk of the BBTools software suite (https://sourceforge.net/projects/bbmap). The raw reads were also filtered to remove contaminating Illumina adaptor sequences and quality trimmed using Trimmomatic v.0.36 (6). The resulting filtered reads were then assembled using SPAdes v.3.13.0 (7). The assemblies were filtered to contain only contigs longer than 500 bp with a k-mer coverage of ≥5×. Genomes containing >500 contigs or an aberrant GC content were removed from further analysis.

Relevant statistics, including GenBank accession numbers and SRA links for each genome assembly, are included in Table 1. The genomes have a mean sequencing coverage of 505× (standard deviation, 152×; minimum, 152×; maximum, 1,437×). The final assemblies have a mean contig count of 48 (standard deviation, 36 contigs; minimum, 11 contigs; maximum, 218 contigs), a mean genome size of 1,725,208 bp (standard deviation, 73,678 bp; minimum, 1,470,770 bp; maximum, 1,997,094 bp), a mean GC content of 30.6% (standard deviation, 0.48%; minimum, 29.9%; maximum, 32.0%), and a mean *N*_50_ value of 161,982 bp (standard deviation, 68,865 bp; minimum, 20,329 bp; maximum, 333,527 bp). Further analysis will reveal the genome dynamics of these important species that cause significant diarrheal disease among humans.

### Data availability.

All data have been released, and accession numbers are listed in Table 1.
